# West Nile virus vector *Culex modestus *established in southern England

**DOI:** 10.1186/1756-3305-5-32

**Published:** 2012-02-09

**Authors:** Nick Golding, Miles A Nunn, Jolyon M Medlock, Bethan V Purse, Alexander GC Vaux, Stefanie M Schäfer

**Affiliations:** 1 Spatial Ecology and Epidemiology Group, Tinbergen Building, Department of Zoology, University of Oxford, South Parks Road, Oxford OX1 3PS, UK; 2 Centre for Ecology & Hydrology, Maclean Building, Benson Lane, Crowmarsh Gifford, Wallingford OX10 8BB, UK; 3 Medical Entomology & Zoonoses Ecology group, Microbial Risk Assessment, Emergency Response Department, Health Protection Agency, Porton Down, Salisbury, Wiltshire SP4 0JG, UK; 4 Centre for Ecology & Hydrology, Bush Estate, Penicuik, Midlothian, EH26 0QB, UK

**Keywords:** Anopheles, Arboviruses, Culex, Culicidae, Disease Vectors, DNA Barcoding, Taxonomic, Introduced Species, West Nile virus

## Abstract

**Background:**

The risk posed to the United Kingdom by West Nile virus (WNV) has previously been considered low, due to the absence or scarcity of the main *Culex *sp. bridge vectors. The mosquito *Culex modestus *is widespread in southern Europe, where it acts as the principle bridge vector of WNV. This species was not previously thought to be present in the United Kingdom.

**Findings:**

Mosquito larval surveys carried out in 2010 identified substantial populations of *Cx. modestus *at two sites in marshland in southeast England. Host-seeking-adult traps placed at a third site indicate that the relative seasonal abundance of *Cx. modestus *peaks in early August. DNA barcoding of these specimens from the United Kingdom and material from southern France confirmed the morphological identification.

**Conclusions:**

*Cx. modestus *appears to be established in the North Kent Marshes, possibly as the result of a recent introduction. The addition of this species to the United Kingdom's mosquito fauna may increase the risk posed to the United Kingdom by WNV.

## Findings

*Culex modestus *is a competent laboratory vector of West Nile virus (WNV, [1]) and regularly bites birds, humans and horses in continental Europe [2]. This mosquito is considered the principle bridge vector of WNV between birds and humans in the Camargue wetland, southern France and is thought to have played a role in the transmission of WNV in the Danube delta, Caspian and Asov sea deltas, and the Volga region in Russia [3]. It has also been implicated in Tahyna and Lednice virus transmission in France and Slovakia respectively [4].

*Cx. modestus *is widely distributed in the Palaearctic region, the larvae inhabit fresh to slightly saline water in irrigation channels, marshes and rice fields [5]. Prior to this report, the only record of this species in the United Kingdom totalled three adults and ten larvae found in and around Portsmouth in southern England in 1944-45 [6].

### The Study

**Figure 1:**
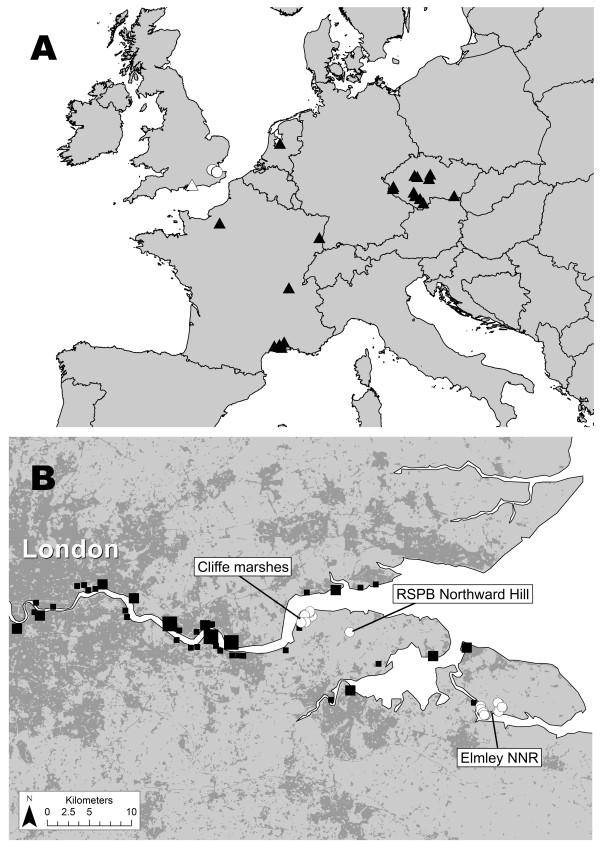
**A) North-west Europe, showing locations where *Culex modestus *populations were detected in this study (white circles), the location of *Cx. modestus *identified in southern England in 1944-5 (white triangle), and recent records from Europe (black triangles; Francis Schaffner, personal communication and articles cited here)**. All of these recent records date from the period 2004-2009 with the exception of the two northernmost French records, which date from 1995 and 1998. B) Thames Estuary area, showing locations where *Cx. modestus *populations were detected in 2010 (white circles) and the locations of international shipping terminals (black squares). To give an indication of the size of the port, black squares are proportional to number of ships arriving during May 2011: small squares 1-25 ships; medium squares 26-75 ships; large squares 75-165 ships. Urban and semi-urban areas, as classified by the United Kingdom Land Cover Map 2000, are coloured dark grey.

Mosquito surveys were carried out during 2010 in the North Kent Marshes, south-east England (Figure 1). Larval surveys were undertaken at two sites - Cliffe marshes (Cliffe; 51°28'58"N 0°28'45"E) and Elmley National Nature Reserve (Elmley; 51°23'03"N 0°47'19"E) - in June, July and August. At each visit larvae were sampled twice using a 1 litre dipper at randomly located points along the edges of drainage ditches, reed beds and pools. A total of 230 points were sampled, across an area of 3.83 km^2^. The relative seasonal abundance of host-seeking female mosquitoes was measured at Northward Hill bird reserve (51°27'45"N 0°33'2"E) using a Mosquito Magnet trap (Liberty plus model, American Biophysics, Rhode Island, USA). This site is 5 km from Cliffe and 18 km from Elmley (Figure 1A). The trap ran for four nights on alternate weeks between April and October.

Larval and adult mosquitoes were identified morphologically using a range of keys [5,7-9]. To confirm the morphological identification DNA barcodes of a subset of *Cx. modestus *specimens from the North Kent Marshes (7 larvae, 10 adults) and the Camargue (3 adults) were generated. A 709 bp fragment of the cytochrome c oxidase subunit I (COI) was amplified by PCR [10] and sequenced. *Cx. modestus *COI barcodes were compared to those obtained from *Cx. pipiens *specimens from the North Kent Marshes (n = 3) and Somerset (n = 6) as well as 35 COI sequences downloaded from GenBank. Phylogenetic analyses were carried out using MEGA5 software [11].

In larval surveys 850 *Cx. modestus *of all stages were collected, along with *Anopheles maculipennis *s.l., *Cx. pipiens *s.l. and *Culiseta annulata*. At both sites *Cx. modestus *was the second most abundant species after *Cx. pipiens *s.l., making up 44% and 23% of the overall larval population sampled at Cliffe and Elmley respectively (Table 1).

**Table 1:** Numbers and proportions (given as %) of larvae collected from Cliffe marshes and Elmley National Nature Reserve

	Month	*Culex* *modestus*	*%*	*Culex* *pipiens s.l*.	*%*	*Culiseta annulata*	*%*	*Anopheles maculipennis s.l*.	*%*
Cliffe marshes	June	0		0		0		0	
	July	131	61	52	24	0	0	31	15
	August	231	38	351	58	0	0	23	4
	
	**Total**	**362**	**44**	**403**	**49**	**0**	**0**	**54**	**7**

Elmley NNR	June	2	3	57	90	1	2	3	5
	July	371	44	408	49	22	3	36	4
	August	120	10	637	52	377	31	91	7
	
	**Total**	**493**	**23**	**1102**	**52**	**400**	**19**	**130**	**6**

**Figure 2:**
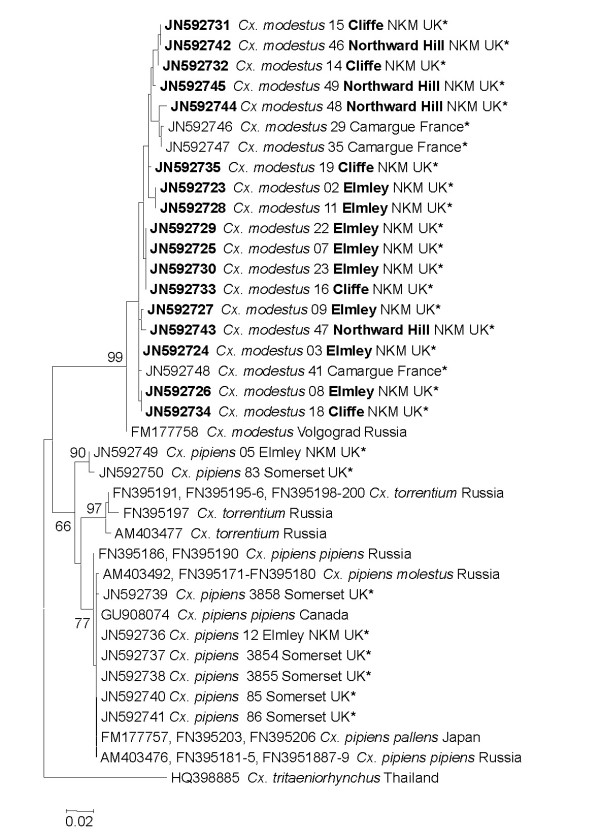
**Phylogenetic maximum-likelihood tree of COI sequences (603 bp) from *Culex modestus *(North Kent Marshes (NKM) specimens in boldface) and other representatives of the *Culex *genus estimated using the T92+Γ+I model of nucleotide substitution, which was selected by MODELTEST**. Bootstrap values are shown for the main clades only. The accession numbers and geographic origin of the 35 GenBank downloads (which represent 10 unique sequences) and COI barcodes generated in this study (asterisked) are shown. Scale bar indicates nucleotide substitutions per site.

A total of 649 adult female *Cx. modestus *were captured at Northward Hill between 12 July and 10 September, with a peak of 325 adults in the second week of August (Table 2). Overall, *Cx. modestus *comprised 75% of the mosquitoes collected at Northward Hill. Morphological identification of *Cx. modestus *was confirmed by DNA barcoding and phylogenetic analyses. All the COI sequences from *Cx. modestus *specimens form a discrete clade with high bootstrap support (Figure 2).

**Table 2:** Numbers and proportions (given as %) of adult female *Cx. modestus *collected at RSPB Northward Hill

	14-18 June	12-16 July	26-30 July	09-13 August	23-27 August	06-10 September	20-24 September
*Cx. modestus*	0	31	272	325	11	10	0
Total catch	0	120	281	350	40	49	21

%	-	26	97	93	28	20	0

### Conclusions

Established populations of *Cx. modestus *have been reported from the Camargue and Dombes wetlands in southern and central France [3,12] as well as in wetlands in the Czech Republic [13] but the species is believed to be more widely distributed than this in Europe. Its previous known northerly limit in Europe was in northern France (see Figure 1A, Francis Schaffner, personal communication) and Oostvardersplassen, the Netherlands [14]. However these records comprise only a few specimens and it is unclear whether there are established populations at these sites. The species was not detected during a recent and intensive survey of the mosquito fauna of Belgium [15]. Our finding demonstrates that established populations of *Cx. modestus *are present in the United Kingdom and provides further support for the existence of northern populations of the species.

It seems unlikely that *Cx. modestus *could have been present in the North Kent Marshes for a long time without being detected. The mosquito fauna of the North Kent Marshes are among the most well sampled in the United Kingdom, both by amateur entomologists and by professionals engaged in mosquito control [16].

An extensive larval survey was carried out at Elmley in 2003 [17]. This survey identified 95 sites containing *An. maculipennis *s.l. but did not detect *Cx. modestus*. In the present larval survey *Cx. modestus *were found to be strongly associated with *An. maculipennis *s.l.; being present in 73% of sites containing *An. maculipennis *s.l. larvae. This suggests *Cx. modestus *was absent from this site in 2003. However the 2003 survey did not record any *Cx. pipiens *s.l. in *An. maculipennis *s.l. positive sites, whilst they were present in 20% of such sites in the present study; suggesting that the sampling strategy employed in 2003 may not have been sensitive to Culicines.

If these *Cx. modestus *populations were established recently, international shipping may well have been the route of introduction. International shipping has previously been implicated in the introduction of mosquito species; including *Cx. modestus *to China [18] and there are a high number of shipping terminals in the area of the North Kent Marshes (see Figure 1B).

A number of vectors and vector-borne diseases have undergone changes in their geographic ranges in recent years, in response to varied biotic and abiotic environmental factors [19]. There is some evidence that *Cx. modestus *is extending its distribution in Europe, with speculation that this may be driven by weather events or changes to wetlands [12,13]. Without detailed information on the previous and current distribution of this species, however, it is unclear what role these factors might play.

A recent review of the potential vectors of WNV [20] concluded that the risk of human cases in the United Kingdom is low due to limited human exposure to potential bridge vectors. However, the risk of transmission of WNV in this part of Kent may be higher than previously supposed, as we have shown that *Cx. modestus *populations exist alongside the potential WNV maintenance vector *Cx. pipiens *s.l. at sites hosting many migratory and resident birds. Since human population numbers in the North Kent Marshes are relatively low and little is known of the dispersal range or host preferences of *Cx. modestus *in the United Kingdom, it is difficult to quantify the significance of any change in risk to humans. It does seem likely, however, that the risk posed to horses, which are often grazed in the North Kent Marshes, will have increased. In light of this, and until the national distribution of *Cx. modestus *is established, surveillance for WNV in the United Kingdom should now focus on this part of Kent.

In summary, the discovery of populations of *Cx. modestus *in southern England suggests a recent introduction of this species and provides further evidence for expansion of its geographic range. There is an associated increased risk posed to the United Kingdom by WNV and other pathogens transmitted by *Cx. modestus*.

## Competing interests

The authors declare that they have no competing interests.

## Authors' contributions

NG designed and carried out the larval surveys, identified the larvae and drafted the manuscript. SMS initially identified the larvae as *Cx. modestus*, carried out the phylogenetic analysis and contributed to revision of the manuscript. JMM and AGCV designed and carried out the adult trapping, identified the adults and contributed to revision of the manuscript. MAN and BVP contributed to the design of the larval survey, interpretation of the results and revision of the manuscript. All authors read and approved the final manuscript.
